# Focal Cortical Dysplasia in an Infant With Aplasia Cutis Congenita: A Case Report

**DOI:** 10.7759/cureus.84842

**Published:** 2025-05-26

**Authors:** Inês Mazeda, Inês Vivas, Sandra Ramos

**Affiliations:** 1 Pediatrics, Unidade Local de Saúde da Póvoa de Varzim/Vila do Conde, Póvoa de Varzim, PRT

**Keywords:** aplasia cutis congenita, brain malformation, epilepsy risk, focal cortical dysplasia, macrocephaly

## Abstract

Aplasia cutis congenita (ACC) is a rare congenital anomaly characterized by a localized absence of skin, most often affecting the scalp. Although often isolated and with a favorable prognosis, it can be associated with other anomalies, including central nervous system malformations. Cortical dysplasia represents a group of disorders caused by abnormal neuronal migration and organization and is a known risk factor for epilepsy. We report the case of a 10-month-old infant with an occipital ACC lesion present since birth. The infant underwent neuroimaging, which revealed a focal area of cortical dysplasia in the right fronto-opercular/insular region. This case highlights the importance of recognizing associated anomalies in patients with ACC and long-term follow-up in the presence of coexisting neuroimaging abnormalities.

## Introduction

Aplasia cutis congenita (ACC) is a rare congenital disorder characterized by the focal or widespread absence of skin present at birth, most commonly affecting the scalp (70-90%), particularly the vertex, but potentially affecting any part of the body, including the face, trunk, and limbs [1,2]. The lesions can vary in size, location, and depth, ranging from superficial epithelial absence to full-thickness defects involving the dermis, subcutaneous tissue, or even bone [3].

ACC has an estimated incidence of 1 to 3 cases per 10,000 live births [1], although the true incidence is likely underestimated, as milder forms may go unrecognized or unreported [2,4].

The etiology seems to be multifactorial, involving both genetic and environmental influences. Suspected exogenous contributors include placental infarction, exposure to teratogenic agents such as methimazole, intrauterine infections or vascular events, fetal trauma, and neural tube defects [1,2,5-7]. The leading pathophysiological hypothesis proposes that mechanical tension during fetal development disrupts the normal process of skin fusion, resulting in localized areas of absent skin [1,3].

The classification system for ACC proposed by Frieden in 1986, widely accepted by both the scientific and medical communities, divides ACC into nine groups according to the anatomical location and distribution of skin defects, associated congenital anomalies, and patterns of inheritance. This classification emphasizes the importance of a detailed clinical evaluation and family history to determine the underlying cause and guide management [1].

While most cases of ACC are benign and isolated, neuroimaging may be warranted when lesions are midline, unusually large, or associated with findings such as macrocephaly or developmental concerns [1]. These features may suggest an underlying structural anomaly, highlighting the importance of a tailored diagnostic approach.

In this report, we present the case of ACC of the scalp associated with focal cortical dysplasia in a 10-month-old infant. This case highlights the importance of recognizing associated anomalies in patients with ACC, as such findings may have significant implications for diagnosis, prognosis, and long-term management.

## Case presentation

We report the case of a previously healthy 10-month-old male infant referred for pediatric neurology evaluation due to macrocephaly. He is the third child of a non-consanguineous couple, with no abnormal family history. Prenatal ultrasounds were reportedly normal apart from increased nuchal translucency. Amniocentesis results were unremarkable. The pregnancy was otherwise uncomplicated, except for maternal anemia treated successfully with iron supplementation. He was delivered at term (39 weeks) via vacuum-assisted delivery, with a birth weight of 3,335 g, head circumference of 36 cm, and Apgar scores of 10 at both 1 and 5 minutes. There were no neonatal complications.

At the time, his head circumference was 48 cm (>97th percentile). The anterior fontanelle was nearly closed, making transfontanellar ultrasound unfeasible. Notably, the father had a head circumference of 59 cm, supporting a probable diagnosis of familial macrocephaly.

**Figure 1:**
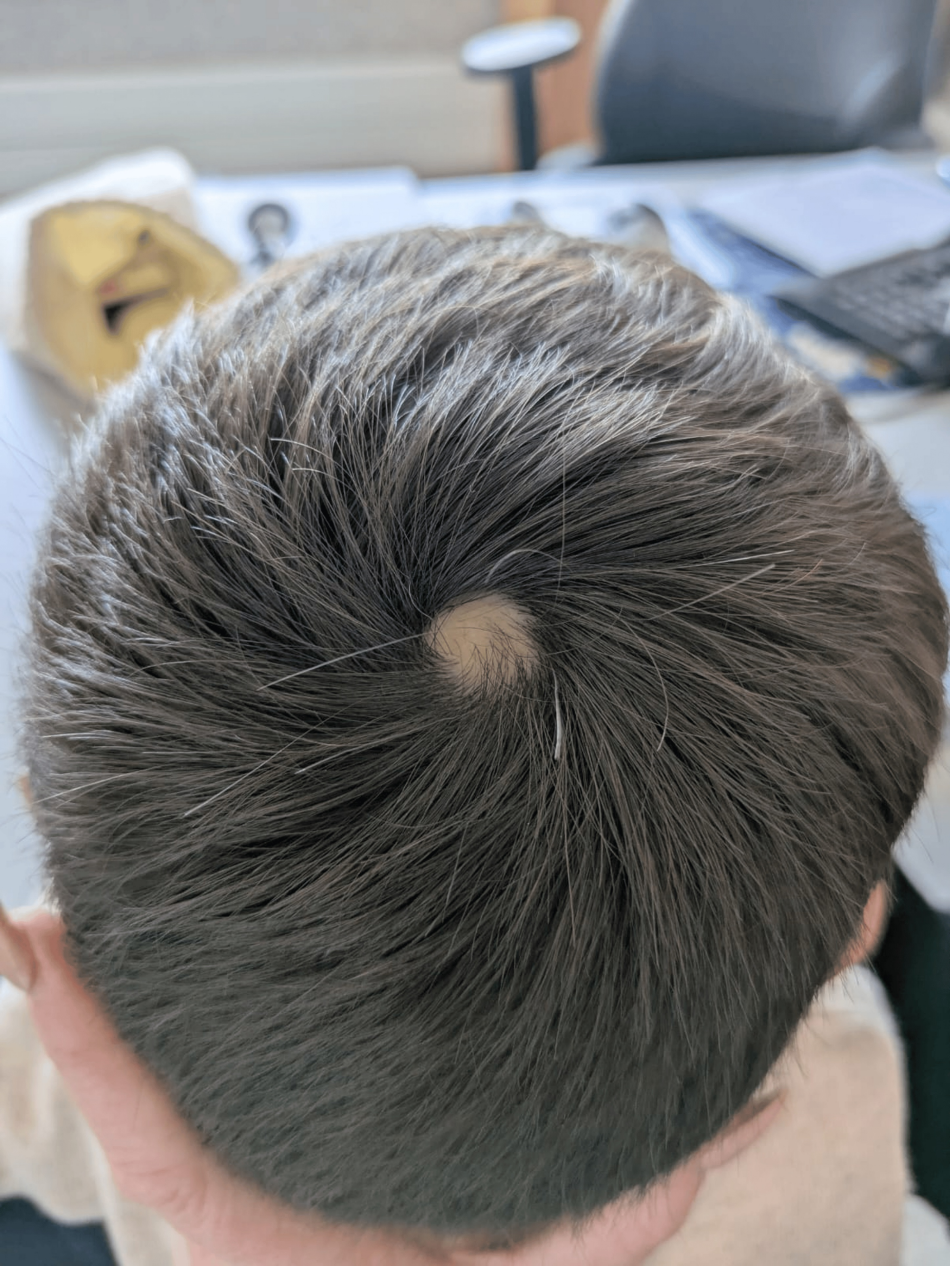
A solitary, well-circumscribed, oval-shaped alopecic scar with a diameter of 15 mm on the vertex of the scalp

A solitary, well-defined vertex scar measuring approximately 15 mm in diameter was noted, present since birth, consistent with ACC (Figure 1). The lesion was cicatricial in nature and had never developed a crust. According to parental report, the lesion was evident at birth and remained unchanged over time. Due to its characteristic appearance and stability, no differential diagnoses were initially raised. Physical and neurological examinations were otherwise unremarkable. Developmental milestones were appropriate for age.

**Figure 2:**
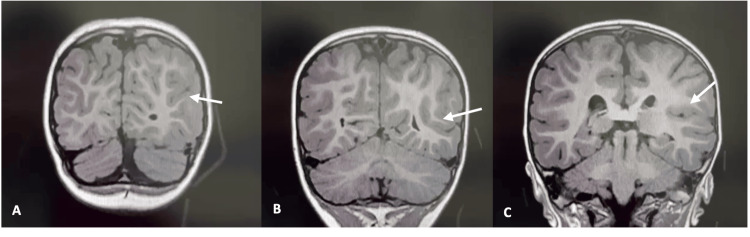
MRI findings of the right fronto-opercular and insular region. (A) Axial MRI slice showing an abnormally deep sulcus in the right fronto-opercular cortex (arrow). (B) Blurred gray-white matter interface in the right insular region, consistent with focal cortical dysplasia (arrow). (C) Enlarged view highlighting atypical sulcal orientation in the right opercular area (arrow).

Due to a favorable clinical course, with age-appropriate neurodevelopment and a sustained yet stable head circumference above the 97th percentile, brain magnetic resonance imaging (MRI) was deferred and performed at 2.5 years of age using T1-weighted sequences. MRI findings revealed a fronto-opercular and insular region (right hemisphere) with an abnormally deep sulcus and atypical orientation. Poor delineation between cortical and subcortical layers was also noted, suggestive of focal cortical dysplasia (FCD). No other structural brain abnormalities were detected. Myelination, corpus callosum, ventricular system, and posterior fossa structures were all normal (Figure 2).

By age 3, his neurodevelopment remained normal, with fluent speech, age-appropriate motor skills, and intact social behavior. The patient never experienced seizures or abnormal movements.

## Discussion

ACC is a rare but well-documented congenital skin anomaly. Although most cases are isolated and benign, ACC may serve as a cutaneous marker for underlying systemic or structural anomalies, particularly involving the central nervous system (CNS), cardiovascular system, or limbs. The diagnosis is clinical, and imaging is generally reserved for cases with atypical features, midline scalp lesions, or associated systemic anomalies [1].

In our patient, a solitary vertex ACC lesion - non-ulcerated, cicatricial, and stable since birth - prompted further investigation in the context of persistent macrocephaly. Although macrocephaly was likely familial, it served as an additional clinical cue to perform neuroimaging, which revealed an unexpected finding: FCD in the right fronto-opercular/insular region.

FCD is a malformation of cortical development that arises due to disruption in neuronal proliferation, migration, or cortical organization during fetal life [8]. It is a well-known cause of medically refractory epilepsy in children and adults [9]. Radiologically, MRI may show blurring of the gray-white matter junction, cortical thickening, and abnormal sulcation - all features present in our case [10].

Although FCD is often diagnosed in patients with epilepsy, particularly in the context of refractory seizures, incidental findings in asymptomatic individuals, such as our patient, have been reported [11]. The clinical implications of such incidental discoveries remain uncertain, as the natural history of asymptomatic FCD is not fully understood. Some individuals may remain seizure-free throughout life, while others may experience delayed onset of epilepsy during childhood or adolescence. In this context, the identification of FCD, even in the absence of neurological symptoms, warrants careful, long-term follow-up, and parental counseling. Surveillance typically includes regular neurological evaluations focused on developmental progress, behavioral changes, and seizure-related symptoms. Routine EEG or imaging is not indicated unless clinical concerns arise. Informing caregivers about early warning signs, such as altered responsiveness, episodic behavioral arrest, or sudden motor events, enables timely medical intervention and may mitigate risks associated with delayed seizure onset [12].

The co-occurrence of ACC and cortical malformation, while rarely reported, has been documented in a few case series and observational studies. According to Frieden’s classification of ACC, our case is best categorized as group IV, which includes ACC associated with underlying embryologic malformations, particularly of the CNS, such as neural tube defects and encephaloceles (Table 1) [1]. Although FCD is not typically visible externally, it is a malformation of cortical development and supports inclusion in this group. Isolated ACC with underlying FCD and no other syndromic features remains a rare and noteworthy association.

**Table 1:** Classification of ACC groups according to Frieden Adapted from Frieden [1] ACC, aplasia cutis congenita

Group	Description	Location	Transmission/inheritance
I	Isolated scalp lesion without other anomalies	Scalp, usually vertex	Sporadic or autosomal dominant
II	Scalp lesion with limb anomalies	Scalp and limbs	Autosomal dominant
III	ACC associated with epidermal or organoid nevi	Scalp, may be asymmetric	Sporadic
IV	ACC overlying embryologic malformations	Abdomen, lumbar skin, scalp; any site	Depends on the underlying condition
V	ACC associated with fetus papyraceus or placental infarcts	Multiple, symmetric areas, often stellate or linear, on scalp, chest, flanks, axillae, and extremities	Sporadic
VI	ACC associated with epidermolysis bullosa	Generalized or scalp	Autosomal dominant or recessive, depending on the type
VII	ACC localized to limbs, without blistering	Limbs	Autosomal dominant or recessive
VIII	ACC associated with teratogen exposure	Scalp (with methimazole); any area (with varicella and herpes simplex infections)	Non-hereditary (environmental cause)
IX	ACC associated with malformation syndromes (e.g., trisomy 13, Adams-Oliver)	Variable	Autosomal dominant or recessive, depending on the syndrome

Importantly, our patient remains neurologically intact at three years of age, with age-appropriate developmental milestones and no history of seizures or behavioral abnormalities. This emphasizes the spectrum of clinical presentation in FCD and the role of ACC as a potential cutaneous marker rather than a predictor of neurological dysfunction.

This case underlines the importance of maintaining a high index of suspicion for associated structural abnormalities in children with congenital scalp lesions. Early neuroimaging can facilitate diagnosis, guide monitoring, and improve prognostic counseling for families.

## Conclusions

This case highlights the potential coexistence of cutaneous and cortical anomalies, even in the absence of clinical symptoms. While ACC is often benign and isolated, its presence, particularly on the midline scalp, should prompt consideration of underlying neurological abnormalities, especially in patients presenting with macrocephaly or other atypical features. The incidental discovery of FCD in our patient emphasizes the diagnostic value of neuroimaging in selected cases.

Given the potential long-term implications of cortical dysplasia, including the future risk of epilepsy, ongoing clinical surveillance is essential. Our patient's favorable neurodevelopmental outcome at three years of age underscores the variability in presentation and prognosis and supports a tailored, case-by-case approach to investigation and follow-up in infants with ACC.
